# Exploring Older Adults’ Pre-Intervention Motivations, Attitudes, Expectations, and Barriers to Participation in the FINGER-NL Lifestyle Intervention to Maintain Optimal Cognitive Functioning: A Qualitative Interview Study

**DOI:** 10.1007/s12126-025-09597-w

**Published:** 2025-04-26

**Authors:** Rebecca Otte, Anja de Kruif, Elke Naumann, MOCIA consortium, FINGER-NL consortium, Marian de van der Schueren

**Affiliations:** 1 Department of Nutrition, Dietetics and Lifestyle, School of Allied Health, HAN University of Applied Sciences, Nijmegen, the Netherlands; 2 Department of Human Nutrition and Health, Wageningen University & Research, Wageningen, the Netherlands; 3 Department of Epidemiology and Data Science, Amsterdam UMC, Location VU University Medical Center, Amsterdam, the Netherlands

**Keywords:** Lifestyle intervention, Prevention, Cognitive decline, Behaviour change, Qualitative design

## Abstract

Multidomain lifestyle interventions hold promise for preventing cognitive decline, but personalized approaches are essential for (maintaining) behaviour change and adherence. The Dutch FINGER-NL trial is based on the Finnish Geriatric Intervention Study to Prevent Cognitive Impairment and Disability (FINGER) and includes 7 lifestyle intervention components, supported by technological elements. This study describes older adults’ motivations and attitudes regarding participation and lifestyle changes at the start of the FINGER-NL trial. This study followed a qualitative descriptive design, using in-depth, semi-structured interviews with 40 purposively selected participants of the FINGER-NL trial. Thematic analysis was applied. For theme (1) 'Reasons to participate', most participants mentioned personal gain, aiming to improve cognitive and physical health. Dementia prevention was a key motivator, driven by concerns about ageing, cognitive decline, and a desire for behavioural change. Public interest and contributing to a broader societal solution were also mentioned. Knowledge about dementia (prevention) was limited, and perceptions were largely shaped by personal experiences of dementia with close ones. In theme (2) 'Contextual factors influencing participation' are discussed, including work, living situation, and health conditions. According to participants, the main 'Lifestyle-related areas for improvement', theme (3), were diet and physical activity, followed to a lesser extent by cognition, sleep, social activities, and stress management. Theme (4) 'Expectations regarding FINGER-NL' discussed barriers to change which included physical health of participants, time constraints, established habits, and financial limitations. Participants emphasized the need for counselling, coaching in diet and exercise, experiencing positive effects of lifestyle change, participation in group setting and practical aspects, such as appointment reminders to support their commitment and adherence to the study. Participants held different experiences and opinions regarding 'Use of technology', theme (5). Personal experiences with dementia strongly influenced the motivation to participate in FINGER-NL, creating urgency for behaviour change. Participants expressed the wish to receive tailored interventions addressing individual needs and circumstances. Longitudinal follow-up within FINGER-NL promises valuable insights for future interventions.

## Introduction

Worldwide aging of the population results in an increase in the prevalence of late-life cognitive impairment and dementia. In the last decade, the number of people affected by dementia increased by approximately 9%, from 47 million people living with dementia in 2015 to around 55 million people in 2019 (Chowdhary et al., 2021). Estimates predict that by 2050, nearly two billion people globally will be aged 60 or older, with dementia prevalence rising from 78 million in 2030 to 139 million in 2050 (World Health Organization, (2021).

The World Health Organization (WHO) has declared dementia a public health priority, focusing on risk reduction and prevention in the absence of curative treatments (Chowdhary et al., 2022). Multiple longitudinal cohort studies have delineated a group of potentially modifiable risk factors and a second group of non-modifiable risk factors (e.g. age, genetic factors). In the recent publication by Livingston, 14 modifiable risk factors were presented, which are hypothesized to collectively account for approximately 45% of dementia cases globally (Livingston et al., 2024). Krivanek et al. (2021) showed that there is growing consensus that behavioural and lifestyle changes focussing on these modifiable risk factors can mitigate cognitive decline and decline dementia risk.

The Finnish Geriatric Intervention Study to Prevent Cognitive Impairment and Disability (FINGER) was the first long-term randomized controlled trial (RCT) aimed at assessing a multidomain approach to prevent cognitive decline (Ngandu et al., 2015). In this large RCT, participants in the intervention group received an intensive multidomain intervention while participants in the control group received regular health advice (Kivipelto et al., 2013). Although effect sizes measured were small, findings of the FINGER study suggest that a multidomain lifestyle intervention can indeed improve or maintain cognitive functioning in older adults from the general population who are at risk of developing dementia (Solomon et al., 2021).

Following the promising results of the FINGER study, the World-Wide FINGERS (WW-FINGERS) network was created (Kivipelto et al., 2020). In the Netherlands, FINGER-NL was established, based on the FINGER principles of the multidomain lifestyle intervention with some extra trial design elements included, including the use of a hybrid intervention components, combining both on site and online meetings (Deckers et al., 2024). The urgency of dementia prevention in the Netherlands is shown by the number of dementia cases in the Netherlands which have increased sixfold; from 50.000 cases in 1950 to 290.000 currently. Dementia, in particular Alzheimer’s disease, has become the fastest-growing cause of death and the healthcare expenses for people with dementia have increased accordingly, from 8.6 billion in 2017, to 10.6 billion in 2020 (Alzheimer Nederland, 2021). However, research among the Dutch general population indicated that there is insufficient knowledge, beliefs and attitudes towards dementia and its prevention to effectively support dementia risk reduction (Vrijsen et al., 2021).

While previous multidomain lifestyle interventions have shown promising results in reducing dementia risk, it will not result in substantial difference unless individuals maintain their behavioural change, which is often challenging (Coley et al., 2019a, 2019b); Krivanek et al. (2021). Motivation is a key factor in adherence to these interventions and influences their overall effectiveness, but the factors influencing motivation are difficult to distinguish and much remains to be explored in understanding its relationship with actual behaviour. One important influence is knowledge about dementia risk reduction and the potential for prevention through modifiable risk factors; understanding that prevention is possible, can influence how individuals adopt and prioritize behaviour change (Meng et al., 2022). 

Personalized interventions, which take into account individual needs and risk factors, are likely more effective compared to general recommendations in promoting sustained preventive health behaviour (Jinnette et al., 2021). Specifically for lifestyle interventions focussed on prevention of cognitive decline and dementia, a personalized approach involves tailoring the activities, advice and approach within the intervention to an individuals’ specific characteristics and needs (Coon & Gómez-Morales, 2023). Despite the promise of personalized approaches, there is still much to learn about older adults’ motivations and expectations regarding such interventions. Understanding their perspectives can help in refining the design and recruitment strategies of future trials. This could, in turn, lead to more valid findings and improve the effectiveness of interventions at an individual and population level (Coley et al., 2019a, 2019b; Kulmala et al., 2021).

The present qualitative study aims to obtain more insight into older adults’ motivation for participating in the FINGER-NL lifestyle trial focused on maintaining optimal cognitive functioning. It also explores their understanding, attitudes, needs and barriers regarding lifestyle and behavioural changes as preventive measures. Additionally, the study discusses participants’ lifestyle habits at the start of the intervention, with a focus on the areas they identified for improvement.

## Methods

### Design

To explore the participants' opinions and narratives, a qualitative design was chosen, in which semi-structured in-depth interviews were planned with selected participants from the Dutch FINGER-NL study (see Box 1). This qualitative design provided the opportunity to explore topics and directions by using the participants' own words to give meaning to their point of view (Queirós et al., 2017). The interview study was conducted independently and parallel with participation in the FINGER-NL study; dropout from the interviews did not have any consequences for participation in the FINGER-NL study and vice versa.

**Table:** 

**Box 1** Study design of FINGER-NL based on Deckers et al. (2024)
FINGER-NL FINGER-NL is a multi-center, multidomain lifestyle intervention trial among 1,210 adults at risk of cognitive decline. The intervention study takes place at five different sites spread out over the Netherlands: Maastricht University (MU), Radboud University Medical Center (RUMC), University Medical Center Groningen (UMCG), Amsterdam University Medical Center location VUmc, Wageningen University & Research (WUR). This study received ethical approval from the Medical Ethical Committee at Amsterdam University Medical Center (UMC) (METC number NL77242.029.21) Recruitment took place in 2022–2023 through the Dutch Brain Research Registry (DBRR, in Dutch “Hersenonderzoek.nl”), using methods such as Facebook advertisements and appearances on national television. Registrants complete a basic questionnaire regarding personal, health, and lifestyle information. Based on this, DBRR automatically prescreens for eligibility and sends a nonbinding study invitation via email. Interested individuals can respond directly, after which researchers contact them for further screening by phone to verify study-specific inclusion and exclusion criteria (Waterink et al., 2025). The participant pools from the different study sites were also used, in addition to recruitment through local databases and (local) newspapers. The inclusion criteria were as follows: (1) age between 60 and 79 years at pre-screening; (2) adequate fluency in Dutch to understand the informed consent and complete study questionnaires; (3) informed consent to all study procedures; (4) Internet access at home; (5) presence of at least three self-reported risk factors for cognitive decline, including a minimum of two modifiable risk factors and one non-modifiable risk factor. Modifiable risk factors were based on self-reported presence (from a single question), while non-modifiable factors included items like family history and subjective cognitive decline (Deckers et al., 2024) Eligibility was verified during a telephonic prescreening and after the baseline visit, participants were randomized in a 1:1 ratio to two groups: the high-intensity group (HI-group) versus low-intensity group (LI-group). Double-blinding was pursued: group allocation was not disclosed to participants. Participants of the HI-group received a personalized, supervised multidomain intervention for 24 months, consisting of hybrid (online and at study site) group meetings and individual sessions focused on a combination of seven lifestyle components; physical activity, cognitive training, cardiovascular risk factor management, nutritional counseling, sleep counseling, stress management, and social activities and a nutritional product (Souvenaid®) that was designed to help maintain cognitive functioning. Furthermore, the HI-group participated in individual sessions guided by a trained lifestyle coach and had access to a digital intervention platform (Ivido) offering custom-made training materials and selected lifestyle apps. The LI-group received monthly online lifestyle-related health advice via the digital intervention platform, Ivido. Primary outcome is 2-year change on a cognitive composite score covering processing speed, executive function, and memory (Deckers et al., 2024)

### Study Population

During the baseline visit for FINGER-NL, participants were informed about the qualitative interview study and asked for permission to be contacted by one of the researchers. Almost all participants gave their consent to be contacted for the qualitative study.

Purposive sampling was conducted among those who had consented, with regard to gender, age, study site and time of registration in the FINGER-NL trial. Study site was considered, as trial sites span across the Netherlands, potentially resulting in varying participant experiences and backgrounds depending on place of residence. Because the recruitment phase of the FINGER-NL trial spanned approximately a year, sampling ensured that participants from various registration periods were included in the interview study as moment of registration might have influence on their reasons for participation and motivation.

### Data Collection

Of all FINGER-NL participants who provided permission to be contacted, a total of 41 were invited for semi-structured interviews within a month of their baseline visit. It is important to note that the interviews took place after the baseline measurements but before the start of the FINGER-NL intervention Programme. Participants from different groups had not yet received study information and therefore the perceptions of the participants could not have been influenced by the randomization process. All participants agreed to take part in this qualitative study and were interviewed by one of the researchers. Out of these 41 interviews, 26 interviews were conducted with participants from the structured intervention group (high-intensity group) and 15 interviews with participants of the self-guided control group (low intensity group).

Semi-structured interviews were conducted using a topic guide based on existing knowledge and literature research (Green & Thorogood, 2018). Topics covered various areas of interest, including the reasons for participation in the FINGER-NL trial, general perspectives on health, knowledge, attitudes and beliefs towards prevention of cognitive decline, and barriers and needs with regard to participation in FINGER-NL. Moreover, one of the topics specifically explored the use of digital aids and technology within the intervention, as this was one of the differences between FINGER-NL and the original FINGER study. The interview guide was reviewed by a team of researchers with expertise in nutrition, health and qualitative research. After conducting 10 interviews, the topic list in the interview guide was reviewed, and no adjustments were deemed necessary. Sample and probing questions were developed for the topics to guide the interview, allowing probing for further information and clarification when appropriate. Examples of some of the topics and probing questions can be found in Table 1. A more elaborate topic guide was used during the interviews. Before each interview, participants were provided with a brief explanation of the research’s purpose and nature.

**Table 1:** Example questions interview

Topic	Example question interview guide
Enrolment	How did you hear about the FINGER-NL study? What are the reasons for you wanting to participate?
Lifestyle	What entails ‘lifestyle’ for you in daily life? Are there things in your current lifestyle that you would want to change?
Prevention	In your opinion, what do you think can contribute to cognitive decline?
Ageing	What is important to you when you get older?
Participation	What are your expectations of the FINGER-NL study? Do you see any barriers to your participation in the coming two years? What would help you during your participation in the FINGER-NL study?
Digital aids	How do you make use of technology in your daily life? Do you use technology to support your health-related behaviours?

Topics were explored by open-ended questions. Participants were encouraged to freely develop the conversation, while the researcher kept the interview in-topic and ensured that all topics from the topic list were sufficiently covered during the interview. Based on the preference of the participant, the interviews were conducted either at the participant’s home or at the research department of the university. One participant preferred an online interview.

### Data Analysis

The interviews were recorded using a digital audio recorder with the permission of the participants and stored on a secured research drive, only accessible by the research team. All interviews were transcribed verbatim using AmberScript (transcription tool) and coded in Atlas.ti (version 23). A thematic approach was used to analyse the transcripts of the interviews. Transcripts were analysed by 2 researchers (RO and AdK) based on the thematic analysis approach of Braun and Clarke (2006), and Braun and Clarke (2019). To increase validity, RO and AdK independently familiarized themselves with the data, closely reading and conducting open coding the same two transcripts to develop a shared understanding and an initial coding framework. This initial coding framework was used to code the remaining interviews, meanwhile extending the code list with additional codes when needed. Through axial coding the related codes were grouped into clusters, forming potential themes, which were organised into a mind map. After checking the potential themes in relation to the coded excerpts and transcripts and refining the essence of each theme, the researchers agreed upon clear definitions and names for each theme. Furthermore, through constant comparison, similarities and differences within and across respondents were identified through selective coding. Finally, the themes and preliminary conclusions were discussed by the researchers and underpinned by relevant examples and quotes from the transcripts (Vaismoradi et al., 2013).

### Ethical Approval

Ethical approval specifically for this qualitative study was granted by the Ethical Research Committee (ECO) of the HAN University of Applied Sciences, Nijmegen, the Netherlands, (ECO 310.12/21). The study was declared not to be subject to the Medical Research Involving Human Subjects Act (WMO), and it underwent examination to ensure compliance with scientific and ethical standards. Potential participants were informed about the aim and procedures of the interview and their right to withdraw from the interview study before, during and after data collection. All participants signed informed consent before the start of the interview and were aware that their anonymized quotes would be used for this publication.

## Results

### Participant Characteristics

Participants’ characteristics are summarized in Table 2. Interviews lasted between approximately 30 min and 75 min; the average interview duration was around one hour. Forty-one participants were interviewed, however, one of the interview audio recordings was of insufficient quality, rendering it unsuitable for transcription and analysis; data from this particular participant were excluded from the final analysis.

**Table 2:** Participant characteristics

Characteristics	N	Mean/Count
Age (years)	40	66.6 (range 61–78 years)
Group	40	High intensity: 24 Low intensity: 16
Sex	40	Male: 14 Female: 26
Working situation	40	Retired (not working): 25 Working: 15
Relationship status	40	Married/in a relationship: 29 Living alone: 11

### Themes and Subthemes

**Fig. 1:**
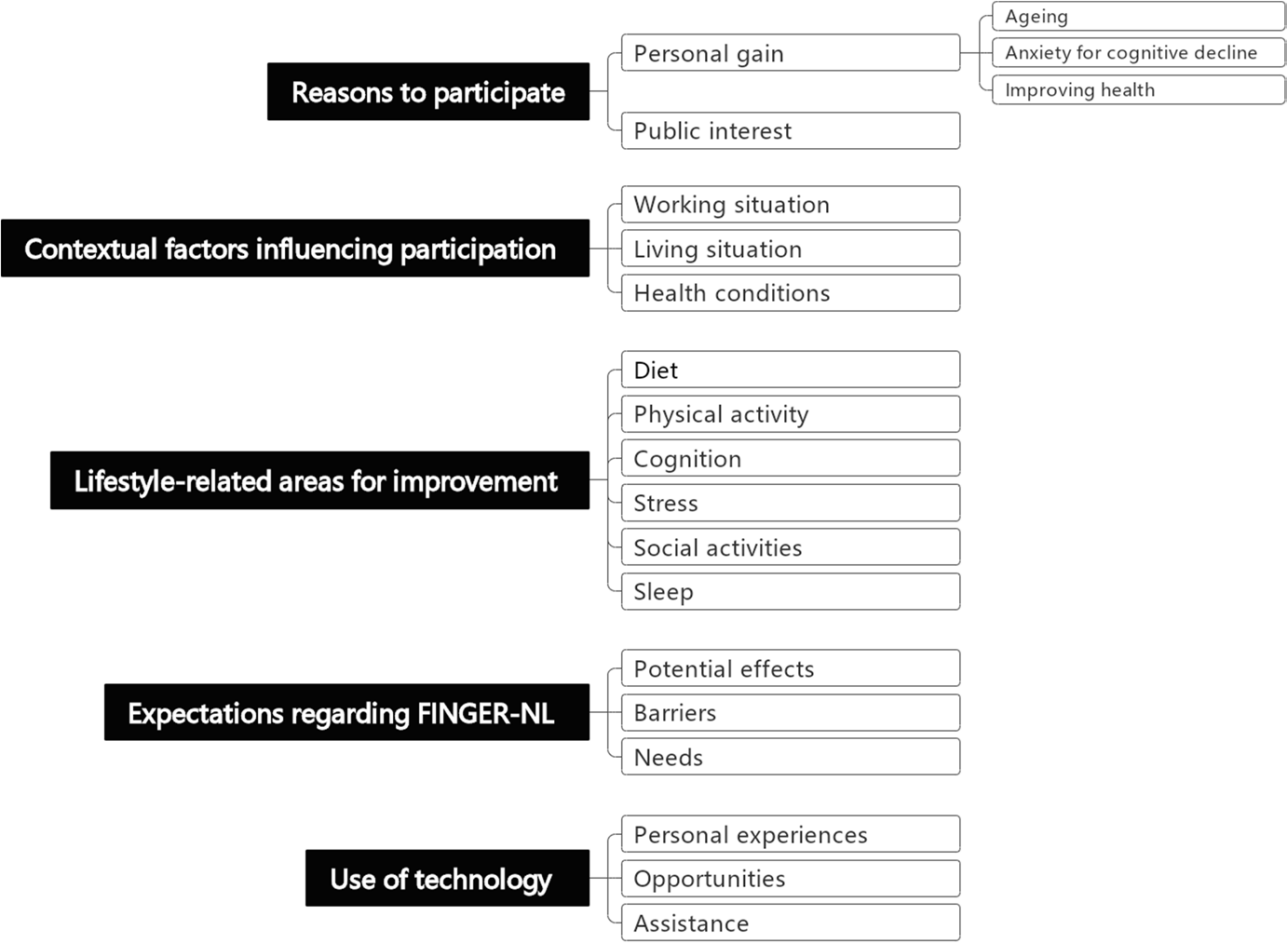
Overview of all themes, subthemes and sub-subthemes

Thematic analysis revealed four main themes important in relation to the research aim. Theme (1) Reasons to Participate discusses the two main reasons participants gave for joining the trial: personal gain, focused on beneficial effects on physical and mental (brain) health. Theme (2) Contextual Factors Influencing Participation explores participants’ working and living situations, as well as their health conditions and the perceived impact on their participation. In Theme (3) Lifestyle-Related Areas for Improvement, participants mainly focused on diet and physical activity, which were mentioned by almost all participants, while other lifestyle factors—cognition, stress, social activities, and sleep— were mentioned less frequently. Theme (4) Expectations Regarding FINGER-NL captures participants’ hopes for potential effects, as well as their perceived barriers and needs. Theme (5) Technology involves participants ‘opinions, experiences and need for assistance regarding the use of technology within a lifestyle intervention aimed at preventing cognitive decline. Figure 1 provides an overview of all themes, subthemes and sub-subthemes.

## Reasons to Participate

The theme discussed two subthemes, each representing distinct reasons participants gave for their motivation to participate: first, personal gain, where participants expressed a desire to improve their health, influenced by their perceptions of ageing and anxiety about cognitive decline; and second, public interest, where participants felt motivated by the opportunity to contribute to research and help others.

### Personal Gain

When asked about their reasons for joining the FINGER-NL trial, the majority of participants indicated that their primary motivation to participate was driven by personal gain; they hoped that participation would bring benefits for themselves, like improving their health.

#### Ageing

When discussing their participation in the FINGER-NL trial, many participants mentioned ageing as a central consideration, with several expressing the hope that the trial would assist them in managing the challenges of getting older. ‘Ageing*’* includes a range of considerations expressed by the participants. Most were in a phase of acceptance and indicated that ageing was a natural process in life. Some experienced life differently now they were older; they mentioned being more able to reflect and putting things into perspective. The majority of the participants indicated that they noticed experiencing various declines, both physical and mental, in recent years as part of their ageing process.

*‘And that, I can already feel that, you know, but then I think: well, I still feel fit and want to keep it that way. But if I start going downhill, well, then I’ll have to accept it. It’s part of life, it’s part of, well, getting older.’ (female, aged 63)*

Additionally, some participants were aware that their condition might not improve anymore and did not look forward to the future.

*‘And I have this idea myself, but it may not be nice to say it, but due to my age, I actually start to live towards my death (…) And I don’t find that very pleasant. Well, I’m declining more and more, physically as well I notice it now. So, but it never gets better. Yes, maybe it gets a little better, but then something else comes up. So, I don’t find the future very appealing.’ (female, aged 71)*

Participants preferred to focus on the present and not think about the future. However, around half consciously engaged with the practical aspects of end-of-life issues. For instance, a few participants mentioned recently moving to an apartment in which in-house care was available if needed, others were sorting personal belongings, making preparations such as creating a living will or discussing euthanasia statements with their general practitioner. Participants emphasized the importance of maintaining control throughout the process of ageing.

*‘But I am working on that, and that is a change because I never thought about it* [becoming older] *before. It never crossed my mind. I mean, everyone will eventually die, but now I think: yes, everyone will eventually die, but I do want to have some control over it. So that is, that is indeed a change.’ (female, aged 72)*

#### Anxiety for Cognitive Decline

Within the context of ageing, the majority of the participants focused on brain health and cognitive state. A relatively high number of participants reported experiencing memory loss recently. This worried them as they were concerned that this might be the first sign of developing dementia, and some participants indicated that this made them realise the need to take action to prevent this from going further. The factual knowledge of participants about the onset of dementia and cognitive decline was limited. Participants indicated that they did not know exactly what the first signs of dementia or Alzheimer’s disease are, and they generally had limited knowledge about the physiology and development of the disease, sometimes resulting in misconceptions thereof.

A vast majority of the participants reported having a close family member diagnosed with dementia, specifically Alzheimer’s disease. Many of these participants were actively involved in the care for their parent(s) with dementia. Their firsthand emotional experiences with dementia very much shaped their perspective and concerns. One participant took a commercial DNA test to check her risk of dementia. The results showed a high genetic risk, which made her feel both thankful for her current health and worried about the future. The majority of participants expressed concerns about their own risk of developing dementia, describing it as a source of anxiety for themselves and worries for the potential effects on their partners and/or children. These concerns were often mentioned as a driving factor in their decision to participate in the trial.

*‘Well, I think, I find it really the scariest prospect when you grow old is that you will, and yes, there are other things of which I think: well, I hope I don’t have to experience that. But dementia, that seems truly terrible to me. Especially that phase when you’re heading towards it, when you start to notice.’ (female, aged 67)*

*‘I honestly hope that this* [diagnose of dementia] *doesn’t happen to me, specifically that my mind remains healthy. I would prefer to face any other condition, but not this.’ (female, aged 64)*

Not all participants shared this concern. Some participants indicated that they thought that development of dementia is hereditary and they perceived having no influence over it. Others were resigned and preferred to let the future unfold rather than worry about it.

*‘Well, maybe it is already in my DNA, but then I think: what’s the use of knowing all of that? If I end up getting as old as my mother? And in that case, I think, I’ll sign up for it. Yes, I don’t need to know it yet.’ (female, aged 63)*

#### Improving Health

Participants indicated that they aimed to specifically improve their brain health and physical health by participating. They hoped that by making the necessary adaptations and following the recommendations within FINGER-NL they could enhance their (brain) health, or at least sustain their current level, with a strong focus on the prevention of development of dementia.

*‘Yes, and I also expect that with the FINGER-NL study, it’s like things that you already know, but where you just need, in a Dutch expression ‘a kick in the butt’, to actually start doing them, because it is clear that it works positively. Yes, that motivates because it is good. My mother is 87, has dementia, and is currently deteriorating rapidly. So yes, you just want to prevent that for yourself.’ (female, aged 61)*

*‘So because I think that by living differently now in a simple way, it can prevent it* [the onset of dementia]*, then I will definitely do that. Yes, so being alert, doing the little quirky things like solving puzzles from the newspaper or whatever.’ (female, aged 61)*

This motivation was also especially strong in interviews with participants struggling with health issues such as diabetes or high blood pressure, as well as those who were overweight and sought to lose weight to achieve a lower BMI. Approximately half of the participants acknowledged being overweight, and even more expressed a desire to lose weight. Some of them already started with a diet or Programme to lose weight, others awaited the start of FINGER-NL.

‘*Well, let me put it this way; I could still lose some weight. I don’t think I’m overweight, but I always have… I think, but that argument is used more often, I also have quite a bit of weight because of the muscles I have, but I haven’t been able to lose those extra pounds, but I am not very strict about it. But if I could lose a few more pounds, that would be good.’ (male, aged 63)*

Almost all participants were aware that participating in the FINGER-NL trial meant that they probably would (have to) change their behaviour particularly with regard to their lifestyle, to obtain effect.

*‘I still want to live for another 30 years or so. Yes, maybe that’s exaggerated, but well, I always say 94, so something needs to change, I think.’ (female, aged 67)*

### Public Interest

A minority of participants mentioned that their decision to participate was mainly motivated by their desire to contribute to public interest. They hoped that their involvement in FINGER-NL would ultimately lead to results which would have broader societal implications by advancing the understanding of dementia prevention. Through their participation, they hope to contribute to the well-being of future generations.

*‘Well, I do want to make a difference for society.’ (male, aged 70)*

The participants’ motivations for personal gain and public interest are not mutually exclusive and some participants also indicate that the two factors both played a role:

*‘Well, I mean, if you can help… Ultimately you hope that you can make a difference, and let’s be very honest, then you especially hope, I think, mainly for my own children and grandchildren. Overall, the general interest is quite important and the grief* [caused by dementia] *is immense for everyone involved.’ (female, aged 64)*

## Contextual Factors Influencing Participation

This theme discusses, from the viewpoint of participants, how contextual factors influence their ability to take part in the FINGER-NL trial. It includes subthemes such as their working and living situation, including employment and relationships, as well as their health conditions.

### Working Situation

The current working and living conditions of participants impact their lifestyle and participation in FINGER-NL. Out of the 40 participants included in this interview study, 25 were retired or not working, while 15 participants were still employed. Among the retired participants, six participants had been declared unfit to work, either temporarily or placed on early retirement. Participants emphasized that their current working situation has an impact on their participation in the FINGER-NL trial. Each participant viewed retirement differently. For some participants, retirement was a recent transition; one participant mentioned having retired just a month ago, while others, like one participant of 78, were already accustomed to the retired lifestyle. Participants shared contrasting experiences regarding retirement. Some expressed a sense of nostalgia and missed the purpose that working provided;

‘*Sometimes I do miss working, Yeah, just missing, missing of being active and engaged and now no one is waiting for you. You don’t have to do anything anymore.’ (female, aged 67)*

*‘When I stopped working, yeah, I simply worked my entire life and then, um, so I fell, I fell into a void and actually, that void is still there.’ (female, aged 71)*

On the other hand, participants enjoyed the freedom and relaxation in their retirement. Participants indicated that they spend their free time differently; some embraced new hobbies, such as community gardening, beekeeping, sports, or voluntary work, including initiatives like the food bank. Some found their retirement days just as busy as before, with all kinds of activities. Some participants decided to go traveling, for example by camper, for longer periods of time and others were involved in babysitting their grandchildren. Several participants indicated that, thanks to their retirement, they now had the time and flexibility to participate in the FINGER-NL trial.

‘*I have always worked with pleasure, well, almost always, I must say, but truly, I have worked with great pleasure 99 percent of the time. But it’s also very nice when you don’t have to work anymore.’ (female, aged 67)*

For the participants who still have a working life, this was experienced a little differently. The majority of these participants work part-time in diverse fields, such as healthcare and consultancy. Most participants indicated that they were happy with their jobs and found challenge and energy in their work. Some participants mentioned contemplating (early) retirement, while others expressed a desire to continue working as long as possible.

*‘Next year I will work a little less, and then the year after that, when I will be 65, then I’ll stop working.’ (male, aged 63)*

*‘As long as I am able to work, I want to keep working.’ (male, aged 63)*

However, these participants were generally somewhat uncertain about whether they could combine work with participation in FINGER-NL. Those with less flexibility in their jobs, in particular, worried that the appointments within the trial might interfere with their work obligations.

### Living Situation

The majority of the participants were in a relationship, most of them being married and living together with their partners. Some participants had experienced the loss of their spouse, while others were not in a relationship at the time of the interview. When asked about their partner’s opinion regarding their participation in FINGER-NL, nearly all participants indicated that their partners were involved and supportive of their decision to enrol. In terms of meal preparation, female participants in relationships indicated that they were generally responsible for cooking dinner, while male participants indicated that their partners primarily provided dinner and they did the grocery shopping. Many participants mentioned having children and grandchildren, generally describing good relationships with them.

### Health Conditions

Participants generally perceived their current health as good, though some experienced age-related issues like arthritis, that limited daily activities. Many shared past health challenges, including cancer, irritable bowel syndrome, Type II diabetes, or heart problems. Common risk factors, such as high blood pressure or cholesterol, were often acknowledged and cited as motivations for joining the FINGER-NL trial, as mentioned under the theme discussing personal gain. A few participants also reported mental health struggles, including depression, PTSD, or burnout. While participants recognised a connection between physical health and dementia, they found it difficult to explain the relationship. One participant also linked mental health challenges to cognitive decline.

*‘Yes, depression shows up in sleep disturbances and losing interest in activities, you end up just watching TV, so yeah, it doesn’t motivate your brain to learn or engage in things. So yeah, over a number of years, it has just gotten worse for me.’ (female, aged 61)*

## Lifestyle-Related Areas for Improvement

This theme focuses on participants’ perspectives on aspects of their current lifestyle that could be improved within the FINGER-NL trial.

During the interviews, participants were asked how they defined lifestyle. While responses varied, the majority of the participants mentioned two key facets: diet and physical activity. Additionally, there was considerable variation, with some participants mentioning sleep or social activities, while others focused on their current lifestyle choices.

*‘What do you mean by lifestyle…? Well, the way of living, and of course, that’s different for everyone, depending where your cradle was, where your bed is and how you live; whether you can live in a healthy environment or an unhealthy environment. Yes, that’s lifestyle, and personally, well, I’m fortunate to have the space, maybe some pollution from traffic, so it may affect my lungs. But where don’t you have that in the world? (…) Yes, when it comes to lifestyle, I believe it means being able to live in a non-stressful, peaceful environment.’ (male, aged 63)*

*‘Lifestyle is simply how you live, whether you eat healthy, sleep well, get enough rest, receive sufficient social input, and engage in regular physical activity.’ (female, aged 64)*

### Diet

Diet was consistently seen as a key aspect of lifestyle, often discussed throughout the interviews. While most participants believed they had a generally good diet, they indicated that they would like assistance in making dietary changes. Participants with type II diabetes were aware of the impact of carbohydrates on their health, while those with high blood pressure aimed to reduce salt intake. Some linked their diet to weight loss and hoped dietary changes would help achieve this. Around half of those who saw a need for improvement mentioned specific areas, such as reducing snacks or treats between meals.

‘*I have a sweet tooth, there shouldn’t be any chocolate near me.’ (male, aged 72).*

Sustainability was brought up by several participants; some mentioned making the switch to using (more) meat replacers or plant-based dairy. Interestingly, participants frequently mentioned their alcohol consumption, recognizing the possible negative impact on their health.

*‘Well, I’m curious about what I can still improve. I always want to continuously improve everything. Honestly, I think I’m doing quite well, but one thing I know, for example, is that I haven’t made the switch to eating less meat and consuming less milk, especially for the sake of sustainability. And yes, for instance, I do enjoy a glass of wine. So, overall, I do have around ten glasses of wine per week. That can be reduced, so I’m trying to do that as well. But well… I also enjoy it.’ (male, aged 63)*

### Physical Activity

Physical activity was frequently discussed during the interviews as having potential for improvement. Participants unanimously recognised the necessity of maintaining regular physical activity as beneficial for their health and well-being. The majority of participants demonstrated conscious decision-making in their approach to physical activity and exercise; some participants walked a lot, alone or in a group, others went grocery shopping by (electrical) bicycle and others engaged in sports such as tennis, yoga, badminton, and swimming. Daily dog walking was commonly mentioned. However, some participants acknowledged a decline in their physical activity levels, particularly after retirement. A few expressed feelings of inactivity or self-described ‘laziness’, but one participant with a background as a personal trainer noted the need to schedule rest periods alongside exercise. Most participants acknowledged that physical activity was less effortless than in their younger years due to increased physical complaints, making movement and motivation more challenging. Participants hoped the FINGER-NL trial would help them become more active, emphasizing the need for tailored approaches, such as outdoor activities, while avoiding activities they dislike, such as indoor gym settings.

*‘And exercise, I have done a lot of sports, and I actually don’t enjoy it at all. But I do hear around me that it is actually very important. When I came across this* [FINGER-NL], *I thought to myself, well, there’s still room for improvement in that aspect for me. So, in that sense, yes, I think and hope that there’s something I could do about it.’ (female, aged 64)*

### Cognition

Participants indicated that they discuss their cognition with friends and family members to check if what they experience is ‘normal’. A small minority of participants were specifically doing activities that were believed to be beneficial for brain health and cognition.

*‘Well, lifestyle is about trying to eat healthy, exercising a lot, especially when you’re retired, and also training my memory, for example, by joining a pop choir.’ (female, aged 69)*

However, the majority of participants indicated that they have little knowledge on the topic and are looking for tools and activities that could effectively stimulate and enhance their cognitive abilities.

### Stress

Stress was mostly mentioned by participants still working, with many reporting it stemmed from a busy schedule or the demands of their job. Some wanted advice on managing stress, while others had accepted it as part of their work responsibilities.

*‘Well, that* [stress] *mostly comes from my work when certain issues escalate and need to be resolved.’ (male, aged 63)*

Only one retired participant mentioned experiencing stress, attributing it to her personality trait of being prone to stress.

### Social Activities

Social activities were part of participants’ weekly routines, including group sports, book clubs, and volunteering. However, some participants, particularly those living alone or without a wide social network, experienced loneliness. These participants indicated that they often chose simpler, less nutritious meals, like soup and bread, instead of more balanced options, because it was ‘no fun’ to prepare meals for yourself.

### Sleep

Sleep was frequently mentioned as an element of lifestyle participants would like to improve, as it impacted many aspects of their lives. Many noted that keeping a sleep diary during baseline measurements made them more aware of their nighttime awakenings. Specifically, several female participants reported a decline in sleep quality during and after menopause, while other participants noted that they needed to go to the toilet more often now that they become older. For some participants, the challenges they face with insomnia left them feeling helpless, as they had made numerous attempts to improve their sleep quality.

*‘I am not a good sleeper, you know. (…) I hope that in this study, they can give me some tips to improve my sleep. I don’t have much confidence in that, though. But well.’ (female, aged 67)*

According to participants, the different aspects of lifestyle are interconnected and influenced each other. Participants emphasized that stress could have a negative effect on their sleep quality. As a result, participants who experienced fatigue and low energy were less likely to engage in physical activity or make healthy food choices.

‘*When I haven’t slept well, then it is important to eat well, but I often lack the energy to pay a lot of attention to that then.’ (female, aged 63)*

## Expectations Regarding FINGER-NL

Participants were asked about their expectations regarding the FINGER-NL trial, which resulted in the following subthemes: the potential effects of the lifestyle intervention on the behaviour and health outcomes, and the identification of both barriers and motivators within the FINGER-NL trial.

### Potential Effects

The participants expressed expectations about the effects of their participation in FINGER-NL, primarily focusing on improvements in mental and physical health. Some specified certain health goals, such as weight loss, lower blood pressure, or reduced cholesterol levels. At the same time, they emphasized the importance of being realistic about the potential effects of the intervention.

*‘Oh, that’s a fun question! Well, I hope that I will be sitting here in two years in the same way. While being in the same condition (…) I won’t become a young girl anymore. But just being able to do everything I’m doing now. (…) Yes, I actually hope to be sitting here in the same way, just two years older, two years further in age.’ (female, aged 67)*

### Barriers

Participants discussed barriers; factors that they perceived as potential challenges or obstacles to implementing lifestyle changes in general. The most frequently mentioned barrier was physical health. Participants indicated that pain, such as back injuries, rheumatoid arthritis or nerve issues affecting their arm and leg movements, significantly hindered their daily activities. Others struggled with balance issues, respiratory problems, fatigue, or the effects of a COVID- 19 infection. A few participants also mentioned that their mental health, particularly depression, could pose challenges to their active participation in the trial.

While physical health was the biggest barrier for most participants, they also identified other barriers. Those with jobs or volunteer work expressed concerns about balancing responsibilities and finding enough time and energy to actively engage in the FINGER-NL trial. Practical challenges, such as planning vacations, were also mentioned. Additionally, participants identified their own old habits as obstacles and were worried that they had to make ‘too big’ adjustments.

*‘What I am afraid of, for example, is having to let go of few small unhealthy eating habits. I try to moderate my alcohol consumption (…) but yes, if in those two years I then also have to stop drinking, I’m a bit afraid that I won’t be able to stick to that.’ (female, aged 69)*

Financial barriers were raised by a few participants, such as the costs of healthy foods, gym memberships, and the reimbursement process for travelling expenses, which could take some time due to administrative reasons. To minimize any potential barriers, participants highlighted the importance of considering the needs of the (elderly) target group in the trial design, such as suitable start times for on-site meetings. They also suggested accounting for personal preferences, like offering options for Non-Western dietary habits in the food diary used to track diets.

### Needs

Participants pointed out the need for advice and guidance to support lifestyle changes. While they valued knowledge, they felt that it wasn’t enough on its own and that they lacked the skills to make changes. They emphasized the importance of active coaching and support for successful engagement and adherence to the intervention.

*‘And I’m someone who wants to do things right. So, if I know that I have to do something, then I’ll do so. And if that accountability is missing, then I think, oh yes, I know myself, so I know I need a ‘big stick.’ (female, aged 67)*

Participants also wanted more insight into the positive effects of their behaviour change, like improvements in blood pressure, to stay motivated. Many would prefer a group setting for the support, possibility to share experiences, and friendly competition it provided.

*‘Yes, and that’s what I’m curious about because I believe that staying in contact with each other also provides more encouragement and motivation.’ (female, aged 70)*

Participants also mentioned practical aspects to meet their needs, such as appointment reminders, providing information on paper for those who prefer it, and the ability to easily contact the research team for assistance when needed. Regular check-ins with the FINGER-NL team and setting small, short-term goals would be beneficial for their motivation, as well as receiving feedback on their progress and efforts.

## Use of Technology

The use of technology was included in the interviews because of its relevance to the FINGER-NL trial in which different technological elements are incorporated. Participants shared their personal experiences with general technology and e-health, as well as the opportunities to use technology in the context of the FINGER-NL trial to change their lifestyle. Also, participants expressed both confidence and uncertainties about their ability to effectively use technology.

### Personal Experiences

Participants’ experiences with (e-health) technology were very diverse, ranging from no experience at all to daily use of technology. Some participants indicated that they didn’t find technology of added value, finding it challenging to see its benefits or relevance in their lives.

*‘Something like a pedometer, for example; it could help, but I don’t see myself constantly paying attention to how many steps I take. Or how much I move, or how much I sit or uhm… No, I believe it would even irritate me after a while.’ (male, aged 68)*

*‘Oh, I don’t want to think about that. No, I got one of those* [smartwatch] *from my children once, but I returned it. That constant monitoring throughout the day, no I really don’t want to think about it. And that you will be monitoring it, what’s happening, or things like that, constantly checking how many steps I take and whether I …. I have a sister-in-law who always says, “I still need to take another 1000 steps” or something like that. I don’t want to think about it. Meddling, no.’ (female, aged 67)*

On the other hand, a smaller group of participants showed enthusiasm for the latest technological developments and considered it a personal challenge to keep up with the technological elements included in the trial.

*‘The developments go really, really fast, and I think that especially my generation has gone from very little to incredible developments. So, what we have experienced is truly enormous, and I realise that I really have to make an effort to keep up with all these things. (…) but I think if you don’t do it and you don’t achieve that, you can’t retrieve it anymore. If older people stop cycling for a long time, they no longer dare to cycle anymore.’ (female, aged 64)*

### Opportunities

When participants were asked about it, they came up with different opportunities they saw in the use of technology within a lifestyle intervention like FINGER-NL. About half already used apps like pedometers, ‘Ommetje’ app which stimulates exercising (Hersenstichting, n.d.), and Strava to track activities. Additionally, some participants used the ‘Eetwijzer’ app from the Dutch Nutrition Centre to track their dietary habits.

‘*Yes, because keeping track has a huge advantage. A) you are aware of what you eat, and B) you are also aware if it when you go way over the line once in a while.’ (female, aged 62)*

Although participants recognised opportunities, some participants expressed concerns about privacy and potential third-party access to their data. Others saw its potential but personally felt that it was not suitable for them.

### Assistance

During the interviews, it was clear that participants had different levels of uncertainty regarding their digital skills. Some participants expressed a need for assistance and found it important that they would have access to help when needed. Others relied on the knowledge and skills of their partners or (grand)children when it came to using technology.

*‘We also have a laptop, but I never use it, my husband always does, so I don’t know, I can use it too. But they will surely tell me how to do it, right?’ (female, aged 67)*

## Discussion

### Summary of Main Findings

The present study, conducted through semi-structured interviews with a selection of participants at the beginning of the FINGER-NL trial, aimed to (1) gain insights into older adults’ motivation for participating in the FINGER-NL trial, (2) investigate their perspectives on lifestyle and behavioural changes as preventive measures for cognitive decline, including needs and barriers, and (3) explore the pre-trial habits of participants related to various lifestyle domains, focusing on areas they aimed to improve.

Participants were strongly motivated to participate in the FINGER-NL study, driven by personal gain and, to a lesser extent, public interest. They aim to improve their mental and physical health, preventing cognitive decline while addressing concerns about ageing and dementia, especially when witnessing dementia in close relatives. Most participants recognised the need for behaviour change, noting the increasing difficulty of behaviour change with age. The working and living conditions of the participants, including retirement status, different daily activities and family relationships, influence their lifestyle and ability to engage in the trial. Participants discussed different aspects of their lifestyle habits, identifying areas for improvement. Lifestyle was primarily defined as encompassing diet and physical activity, with some participants mentioning sleep and social activities. Participants expressed a need for dietary improvements, particularly regarding snacks, treats, and alcohol consumption.

Participants in the FINGER-NL trial expressed their expectations regarding the intervention’s effects on their mental and physical health, including weight loss and improved well-being, while acknowledging the importance of realistic expectations. They also identified barriers like physical health issues, time constraints, and old habits, and motivators such as guidance, feedback on progress, group support, and practical assistance to facilitate lifestyle changes and adherence to the trial. Lastly, participants varied in their experiences with technology and digital skills, with some seeking assistance and relying on family members for technological support.

### Motivation for Participation

Participants indicated that personal benefits were the main reasons for their participation, such as preventing cognitive decline, improving health, and changing behaviour. These motives were stronger than altruistic reasons, like contributing to science or society. This aligns with a previous study of FINGER and the Multidomain Alzheimer Preventive Trial (MAPT) participants, where improving lifestyle was focused on maintaining functional independence, a theme also reflected in our interviews (Coley et al., 2019a, 2019b).

Most participants in our study reported in the interviews that they have or had a family member with dementia, usually a parent, but sometimes also siblings. Family history and hereditary predisposition were profound recurring themes in the interviews emphasized the importance thereof for the participants. Additionally, the prevention of these issues was a key focus for many. This is in line with the research conducted by Anna Rosenberg et al. (2020a, 2020b) which emphasized that family history plays a vital role in shaping what people know and how they feel about cognitive decline and related disorders. Other studies support the findings that indirect experiences, such as having a family member with dementia, are linked to greater awareness and a better understanding of modifiable risk factors, such as the domains addressed in the FINGER-NL trial (Glynn et al., 2017).

Within the FINGER-NL trial, both modifiable dementia risk factors and non-modifiable risk factors, such as family history and concerns about cognitive decline, are used as inclusion criteria (Deckers et al., 2024). Therefore, the study population might not be fully representative of the general population as most participants had firsthand experience with dementia in their family. Motivation to participate in similar trials or to modify one’s lifestyle may vary within a population without direct dementia experience. Nevertheless, the prevalence of dementia suggests that many people in the Netherlands have experienced dementia in their social environment (Alzheimer Nederland, 2021).

### Understanding and Attitudes Towards Lifestyle and Behavioural Changes, Needs and Barriers

Participants indicated that diet and exercise were the main pillars for lifestyle changes, highlighting their importance in the participants’ awareness. Regarding diet, participants acknowledged that alcohol consumption is not beneficial for their health. The use of alcohol at certain times, such as in the afternoon or after dinner, seemed to be a generational habit (Dare et al., 2020), which is further explained by Kersey et al. (2022), who suggest that drinking is influenced by various social and cultural identities. The other domains, such as metabolic and vascular health, sleep, stress, cognition, and social activities, were less directly linked to overall health and cognitive decline. Participants indicated that they mainly lacked knowledge on these topics.

Participants were generally aware of risk factors such as high blood pressure and high cholesterol levels that increased their perceived susceptibility to diseases, but often did not mention the relationship between these factors and their cognitive health. Previous research supports the findings of this study, emphasizing that knowledge about dementia, including (modifiable) risk factors, is generally low, even among individuals with a higher level of education (Pacifico et al., 2022; Vaportzis & Gow, 2018).

Compared to the original FINGER study, the FINGER-NL trial also incorporates online elements and technological support (apps, dietary journals, online coaching). During the interviews, we specifically asked about participants’ views on using technology in a lifestyle intervention. Digital experience was not solely age-dependent. Some older participants (75+) were comfortable using computers, smartphones, and smartwatches, while others in their 60s struggled with technology. In general, research shows that online lifestyle programs have the potential to enhance brain health outcomes and may play a role in dementia prevention (Wesselman et al., 2019).

### Strengths and Limitations

The qualitative design used in this interview study facilitated an in-depth exploration of the experiences and expectations of older adults just before the start of the FINGER-NL trial. Utilizing semi-structured interviews allowed for in-depth discussions of relevant topics (Green & Thorogood, 2018). Participant recruitment included four different sites where the FINGER-NL trial is conducted, encompassing the whole of the Netherlands, from the northern site in Groningen (University of Groningen) to the southern site in Maastricht (Maastricht University). Although there were no striking disparities among participants from the different sites, subtle regional and cultural differences were noticeable, which are characteristic for different parts of the Netherlands (Groenewegen et al., 2003), like the more ‘bourgeois lifestyle’ as described by the participants from the south of the Netherlands. These distinctions could potentially influence aspects of lifestyle, including dietary choices and alcohol use.

### Considerations Regarding Intervention Design and Further Research

Lifestyle interventions have gained increasing attention as effective strategies for addressing a wide range of health conditions, particularly for the prevention of cognitive decline, with multiple global studies currently investigating their effect (Coley et al., 2022; Rosenberg et al., 2020a, 2020b). However, the challenge lies in sustaining long-term lifestyle changes, during, but also after the trial. Therefore, gaining deeper and more nuanced understandingof the factors driving participation in lifestyle related RCT’s is necessary for enhancing the design and recruitment strategies of coming trials. The findings in this study showed the diversity in participants’ levels of knowledge and their practical application of this knowledge in lifestyle related behaviour change. While some participants showed gaps in their knowledge of specific lifestyle domains, others emphasized their need for support in effectively applying the knowledge they had gained or already had. Furthermore, individual differences, for example, in working and living situation, can influence the participation in lifestyle intervention. For example, this research shows that employment status influences the amount of time participants have available for participation, where retired people are less likely to face this time constraint. These differences in knowledge acquisition and individual circumstances highlight the need for tailored interventions that address specific needs and challenges to support healthy ageing (Bukman et al., 2014; Coley et al., 2019a, 2019b; Rosenberg et al., 2020a, 2020b).

To gain a deeper understanding of the changing perspectives of participants in a lifestyle intervention, specifically in the FINGER-NL trial, and to capture their evolving experiences, reflections, and opinions over time, it is necessary to follow participants throughout the two-year trial using a longitudinal study design. This enables us to keep track of participants over the course of the trial by employing semi-structured interviews at different pre-defined timepoints. The participants from our current study will continue to be involved in the interview study as they progress through the FINGER-NL trial, either in the structured intervention group (high-intensity group) or the self-guided control group (low-intensity group), enabling us to examine how group allocation influences their experiences and outcomes in the trial.

## Data Availability

The anonymized data from the interviews that support the findings of this study are available from the authors upon reasonable request.
